# First complete plastomes of the emblematic Andean tree genus *Polylepis* Ruiz & Pav. (Rosaceae)

**DOI:** 10.1038/s41598-025-20603-8

**Published:** 2025-09-23

**Authors:** Jay Edneil C. Olivar, Jana Ebersbach, Alexandra N. Muellner-Riehl, Isabell Hensen, Michael Kessler, Christiane M. Ritz, Marcus Lehnert

**Affiliations:** 1 Department of Molecular Evolution and Plant Systematics & Herbarium (LZ), Institute of Biology, Leipzig University, Johannisallee 21–23, 04103 Leipzig, Germany; 2 German Centre for Integrative Biodiversity Research (iDiv) Halle-Jena-Leipzig, Puschstr. 4, 04103 Leipzig, Germany; 3 Plants and Politics Working Group, Leipzig Lab, Leipzig University, Straße des 17. Juni 2, 04107 Leipzig, Germany; 4 Institute of Geobotany, Martin Luther University Halle–Wittenberg, 06108 Halle, Germany; 5 Department of Systematic and Evolutionary Botany, University of Zurich, Zollikerstrasse 107, CH-8008 Zurich, Switzerland; 6 Senckenberg Museum for Natural History Görlitz, Senckenberg–Senckenberg–Leibniz Institution for Biodiversity and Earth System Research, D-02826 Görlitz, Germany; 7 International Institute (IHI) Zittau, Dresden University of Technology, Markt 23, D-02763 Zittau, Germany

**Keywords:** Andes, Phylogeny, Plastome, *Polylepis*, Rosaceae, Evolution, Genetics, Plant sciences

## Abstract

Plastome genomics and phylogenomics are essential tools for understanding plant diversity and evolution. Here we report the complete plastomes of *Polylepis australis* and *P. microphylla*, two species of the Andean tree genus *Polylepis* which forms the world’s highest treelines, using a whole-genome sequencing approach. The assembled plastomes (ca. 155 kb) exhibit the typical quadripartite structure of angiosperms with highly conserved gene content and organization. A total of 117 unique genes (81 protein-coding, 32 tRNA, and 4 rRNA) were annotated in the assembled plastomes. Comparative analysis revealed significant differences from previously reported partial plastomes of *Polylepis*, which lacked one copy of the inverted repeat (IR) region due to the limitations of long-range PCR methods. Phylogenetic analysis of whole-plastome data of Rosaceae species across subfamilies using Maximum Likelihood (ML) confirmed the monophyly of *Polylepis* and its placement within the tribe Agrimonieae. Our results highlight the reliability of whole-genome sequencing for generating complete, high-quality plastome sequence information. This provides a foundation for broader phylogenomic studies across the genus to test species boundaries, evaluate hybridization events and evolutionary trajectories, and develop conservation and restoration strategies.

**Supplementary Information:**

The online version contains supplementary material available at 10.1038/s41598-025-20603-8.

## Introduction

Chloroplasts are active metabolic organelles responsible for sustaining nearly all life on Earth primarily by converting solar energy to carbohydrates and thereby releasing oxygen through photosynthesis^1^. Advances in sequencing technology have led to the increase in the publication of complete chloroplast genomes (plastomes) which enhanced our understanding of plant biology and diversity, improved quality of crops, and harnessed the potential of chloroplasts for transgenic protein production^1^. Plastomes of land plants exhibit a highly conserved structure and organization. They typically represent a single circular DNA molecule with a characteristic quadripartite structure, consisting of two identical inverted repeat (IR) regions which separate the large single-copy (LSC) and small single-copy (SSC) regions, and usually range in size from 107 kb to 218 kb^1,2^. This highly conserved organization has practical applications. For example, it allows the validation of parental origin and purity in horticultural and crop varieties, the identification of regulatory genes through homology analyses, the development of DNA barcodes for plant identification, and the efficient engineering of transgene cassettes for biotechnological applications^3–5^. In plant systematics, plastome assemblies are indispensable tools in elucidating phylogenetic relationships because of their mostly uniparental inheritance and uniform rates of sequence evolution^6^. Plastome phylogenies, in turn, may facilitate the rapid discovery of potential sources of bioactive compounds and adaptive traits, the clarification of taxa boundaries, and identification of priority areas for conservation^6,7^. A plant genus that has eluded a sound phylogenetic reconstruction so far is the emblematic high Andean tree genus *Polylepis* Ruiz & Pav. (Rosaceae).

*Polylepis* is a genus of ca. 45 wind-pollinated species of shrubs and trees mainly recognized for its multi-layered, shedding barks^8^. The genus is endemic to the central and northern Andes, where it forms loose forests and the world’s highest treelines, and provides critical habitats for numerous Andean plants and animals^9–11^. In addition, *Polylepis* forests provide a range of ecosystem services, including clean water, erosion control, firewood, fodder, medicinal plants, and many more^12,13^. Despite their ‘iconic’ status in the Andes and the range of ecosystem service they offer, *Polylepis* forests are one of the most endangered habitats in the high Andes, having lost 90% of their natural cover over the last two millennia due to human activity and climate change^14–16^. Conservation efforts do exist but they are limited to a few species only^17^. This is partly due to the difficulties in establishing clear species boundaries, which contrasts sharply with the clear delimitation of the genus. Espinoza & Kessler^8^ published the most recent monograph of *Polylepis* utilizing the general lineage concept^18^ combining morphological, biogeographical, and ecological data, but their treatment has yet to be evaluated using molecular data. Previous studies attempting a phylogenetic reconstruction of *Polylepis* relied mainly on nuclear markers but have failed to produce a well-supported phylogeny, attributed to extensive hybridization and gene flow among species and low phylogenetic signal in the data^19–21^. Complete plastome data may therefore help to clarify species boundaries within *Polylepis* by providing a rich source of genetic information capturing phylogenetically informative conserved and variable regions−many of which are undetectable by traditional approaches.

GenBank currently contains four accessions (KY419921.1, KY419989.1, KY419992.1, OQ834952.1) of partial plastomes for *Polylepis* submitted by Zhang et al.^22^ to study deep phylogenetic relationships and diversification history of Rosaceae^22,23^. The plastomes were generated in overlapping fragments using a long-range PCR method^22^. However, this method is known to introduce artifacts such as chimeras, mutations, and heteroduplexes^24,25^. Here we report complete plastome assemblies of *P. australis* Bitter (sect. *Australes sensu* Boza Espinoza & Kessler^8^) and *P. microphylla* Bitter (sect. *Reticulatae sensu* Boza Espinoza & Kessler^8^) using a whole genome sequencing approach. We aim to characterize the plastome of *Polylepis* to provide a foundation for reconstructing the phylogeny of the genus and to support broader taxonomic and evolutionary studies.

## Results

### Plastome assembly and features

**Fig. 1:**
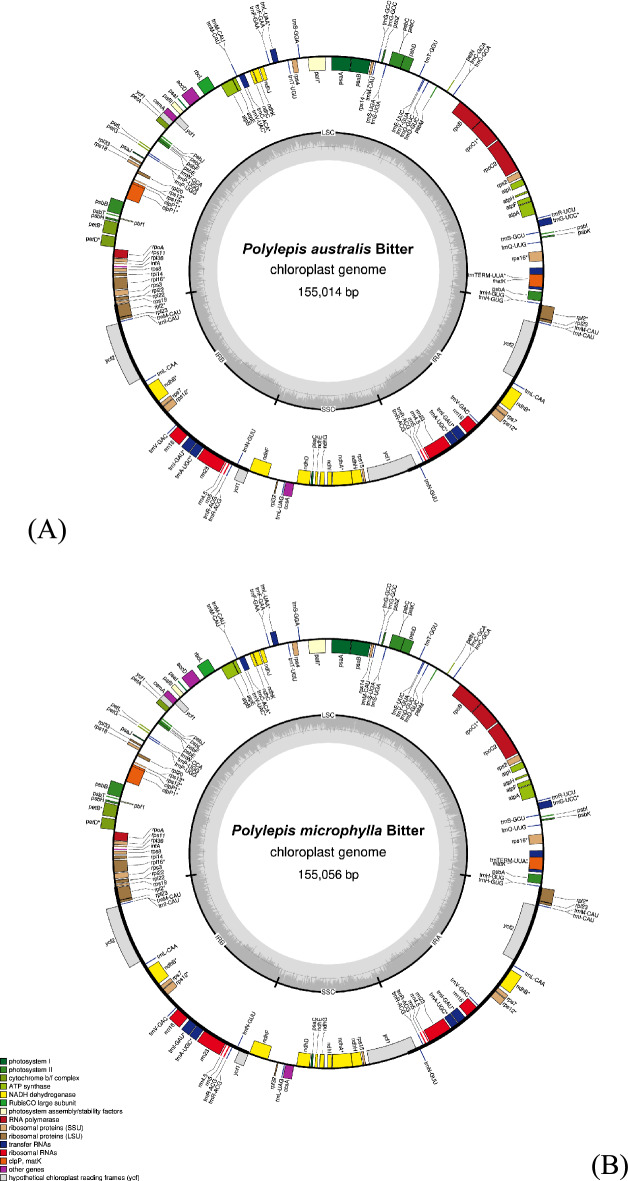
Plastome map of (**A**) *P. australis* and (**B**) *P. microphylla* showing typical quadripartite structure (LSC: large single-copy, SSC: small single-copy, and two IRs: inverted repeat regions). Colored boxes represent genes grouped by functional categories. The innermost grey circle indicates GC content (%). Genes located inside the circle are transcribed clockwise, while those on the outside are transcribed counterclockwise.

Approximately 12.9 million paired-end raw reads were generated on an Illumina platform and after screening ca. 7.6 million reads were successfully mapped with 106X−118X sequencing depth (Table 1). Sequencing depth was high enough to produce high quality plastome assemblies for both *Polylepis* species and with getOrganelle we assembled ca. 155 kb circular plastomes, which is comparable to other species of Rosaceae^25,26^. The assembled plastomes conform with the typical quadripartite structure in angiosperms (Fig. 1) and show comparable GC content (37.3%) with other members of Rosaceae^25,26^. All sequence information of the assembled plastomes is provided in Table 1.

**Table 1:** Sequence information and Illumina next-generation sequencing (NGS) data of the *Polylepis* plastomes.

Species	Raw Reads No.	Mapped Reads No.	Sequencing Depth	Cp Genome Length (bp)	GC Content (%)	LSC (bp; average depth)	SSC (bp; average depth)	IRs (bp; average depth)
*P. australis*	12,889,791	7,629,990	106X	155,014	37.3	85,173; 138X	18,723; 144X	25,559; 280X
*P. microphylla*	12,977,711	7,652,849	118X	155,056	37.3	85,271; 142X	18,693; 124X	25,546; 296X

*LSC (large single-copy region), SSC (small single-copy region), and IRs (inverted repeats region).

**Fig. 2:**
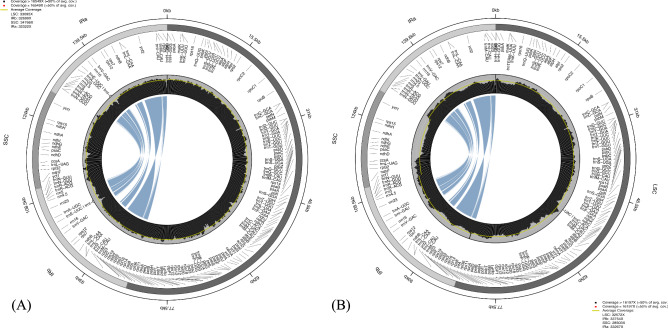
Robustness of plastome assemblies analysed with PACVr for (**a**) *P. australis* and (**b**) *P. microphylla* visualizing coverage depth (center circle) at 250 bp window size and equality (blue lines) of the IR regions. Yellow line inside the inner circle denotes average coverage depth.

The two *Polylepis* plastomes possessed 117 unique genes including 81 protein-coding genes, 32 tRNA genes, and four rRNA genes. Both plastomes are highly conserved in gene type, order, and content (see Supplementary Table 1). Moreover, the resulting assemblies contained no ambiguous bases, suggesting that the plastome sequences are complete and free of unresolved regions. In order to assess the robustness of the assemblies, we used PACVr (plastome assembly coverage visualization in R)^27^ to visualize coverage depth and gene synteny across the IR regions on the circular plastome map. With a window size of 250 bp, all regions are well above the average coverage depth, and the synteny results show equality of genes in the IR regions (Fig. 2) indicating the robustness of the assembled plastomes. We aligned the two assembled plastomes with accessions of partial plastomes of *Polylepis* available in GenBank. Noticeably, the partial plastomes are ca. 20 kb smaller than the assembled plastomes due to the presence of only one copy of the IR region in the partial plastomes. Duplicated regions are often missed by PCRbased approaches because of conserved priming sites. In some of the spacer regions of the partial plastomes, we observed 4–10 bp insertions of tandem repeats, ambiguous and chimeric sequences, which are probably PCR artifacts (see Supplementary Figure 1 & 2). In terms of overall gene order, the plastomes of *Polylepis* species exhibit a highly conserved structure, with no major rearrangements detected among the sampled genomes. The relative positioning of coding regions, tRNAs, and rRNAs is consistent across species, indicating structural stability of the plastome architecture within the genus (see Supplementary Figure 1). The average sequence similarity of the *Polylepis* plastomes is 99.2% (see Supplementary Table 2). Raw sequence reads and annotated plastomes are available on European Nucleotide Archive (ENA) under project accession number PRJEB89853.

**Fig. 3:**
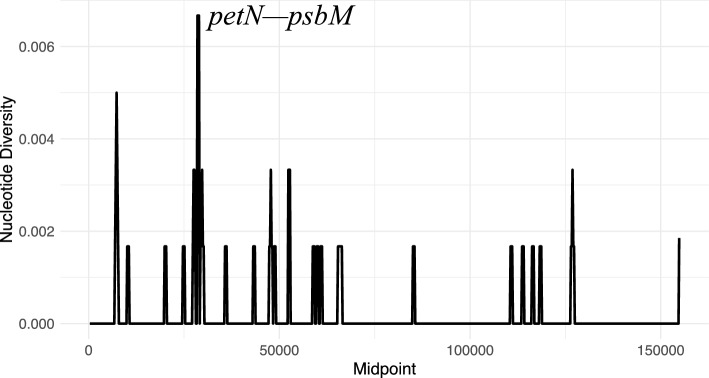
Sliding window analysis of the two assembled *Polylepis* plastomes. The x-axis represents the midpoint position of each 250 bp window, while the y-axis indicates the nucleotide diversity (π) within each window.

Nucleotide diversity (π) was calculated to evaluate sequence variability levels in the two assembled plastomes using DnaSp v6^28^. The values range from 0 to 0.007, and the average value was 0.0002. Only one polymorphic region (π > 0.007) was identified (Fig. 3), this is the spacer region between *petN—psbM* in the LSC. The values reported here are relatively low compared to studies on other members of Rosaceae^29,30^, which may be attributed to the limited sample size (only two samples) and/or the close phylogenetic relationship between *P. australis* and *P. microphylla*. The spacer regions *petA–psbJ, trnT–trnL, trnH–psbA,* and *rpl32–trnL* have consistently been identified as hypervariable loci in plastome assemblies across different Rosaceae genera^29–32^. Notably, the studies reporting these patterns were based on substantially larger taxon sampling—often spanning multiple species—which increases the likelihood of capturing the full extent of sequence variation present in these regions. Such comprehensive sampling can reveal phylogenetically informative polymorphisms that may remain undetected in smaller datasets, thereby providing a more accurate estimate of their comparative utility for barcoding and phylogenetic inference.

### Phylogeny

**Fig. 4:**
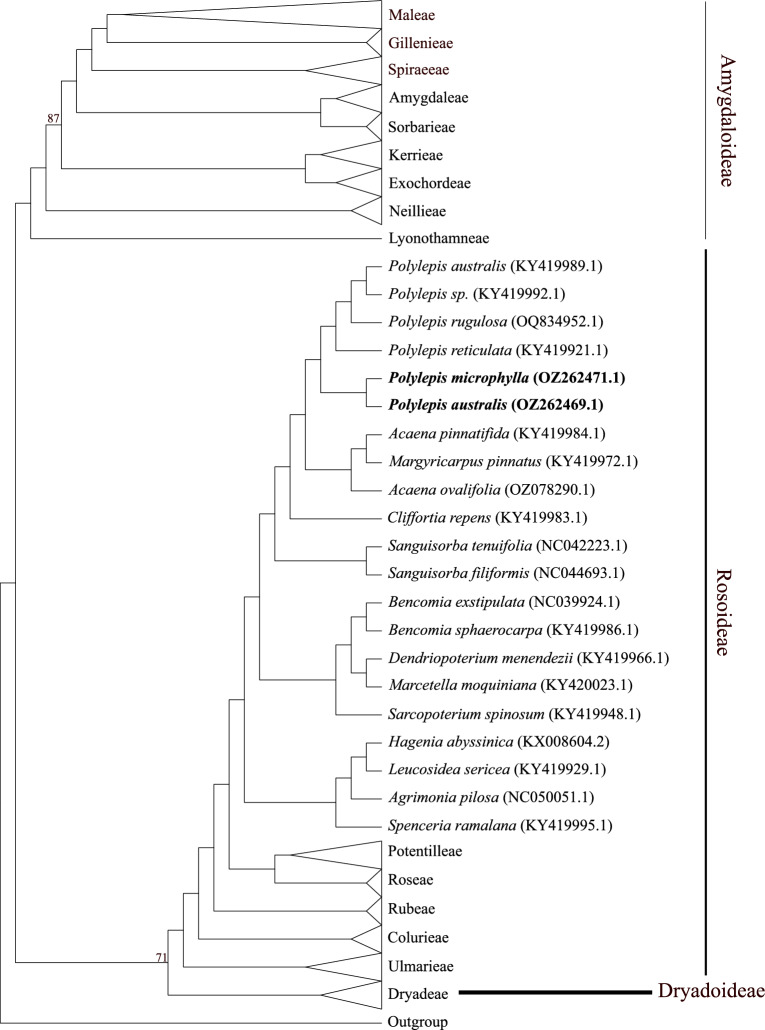
Maximum Likelihood (ML) phylogenetic tree of the family Rosaceae based on plastome sequences. Bootstrap support (BS) values are shown at nodes where support is less than 100. Monophyletic tribes are collapsed, only shown here is the tribe Agrimonieae. Tribal names follow Zhang et al.^22^ and information following species name are GenBank accession numbers. In bold are the newly assembled plastomes of *Polylepis*. The complete tree is available as supplement.

We assessed the phylogenetic position of the assembled plastomes in the family Rosaceae using Maximum Likelihood (ML) analysis. The ML tree (Fig. 4) is well resolved and shows overall high bootstrap (BS) support values. The *Polylepis* accessions formed a strongly supported (BS=100) monophyletic group. A partial plastome accession of *P. australis* is available in GenBank; however, it is not in monophyly with our assembled *P. australis* plastome. This discrepancy could be possibly due to the absence of the second IR region in the partial plastome sequence. The *Polylepis* clade is sister to the *Acaena*—*Margyricarpus* clade (BS=100) and is nested in the tribe Agrimonieae (BS=100). All tribes were resolved as monophyletic. Some genera with multiple accessions were not resolved as monophyletic, which may be attributable to sampling biases or outdated taxonomic identification of the accessions (see Supplementary Figure 3 for complete ML tree and see 10.5281/zenodo.17077866 for alignment). It is essential to revisit the source material (e.g., voucher specimens and living collection) of these accessions to make accurate assessments of generic affiliations and monophyly. We also conducted a ML analysis restricted to *Polylepis* and two outgroup taxa, with the inverted repeat (IR) regions excluded (see Supplementary Figure 4). The resulting topology was consistent with that obtained from the full plastome dataset, suggesting that the observed signal is robust to the exclusion of duplicated regions. However, the placement of *P. australis* partial plastome warrants further scrutiny. One possibility is that the *P. australis* accession was misidentified, in which case the source material should be revisited to confirm its taxonomic identity. Alternatively, the pooled PCR strategy used to generate the partial plastome sequences may have introduced assembly artifacts. To resolve this issue, comparisons with independently generated datasets—such as those produced using long-read sequencing technologies—will be critical. The subfamily Dryadoideae is resolved as sister to subfamily Rosoideae, consistent with the plastome phylogeny of Zhang et al.^22^. This contrasts with the nuclear genome phylogeny of Xiang et al.^31^, which resolves Dryadoideae as sister to the remainder of the Rosaceae.

## Discussion

The complete plastome assembly of *Polylepis australis* and *P. microphylla* generated in this study represent the first fully assembled chloroplast genomes for the genus. These plastomes exhibit the typical quadripartite structure of angiosperms, with comparable genome sizes (ca. 155 kb) and GC content (37.3%) to other members of the Rosaceae, consistent with findings from previous studies^22,25,26,31^. The high sequencing depth and uniform coverage across all regions, including the IR regions, support the robustness and completeness of our assemblies. Gene content and structure are highly conserved between the two *Polylepis* species, consistent with other genera in Rosaceae except for the genus *Filipendula* which exhibits structural differences, including gene loss, transposition, and inversion^26^. When aligned with already available partial plastomes of *Polylepis*, our complete assemblies are ca. 20 kb larger due to the absence of one IR region in the partial assemblies. This underscores a common limitation of long-range PCR methods, which often fail to amplify duplicated regions like IR primarily due to conserved priming sites.

**Fig. 5:**
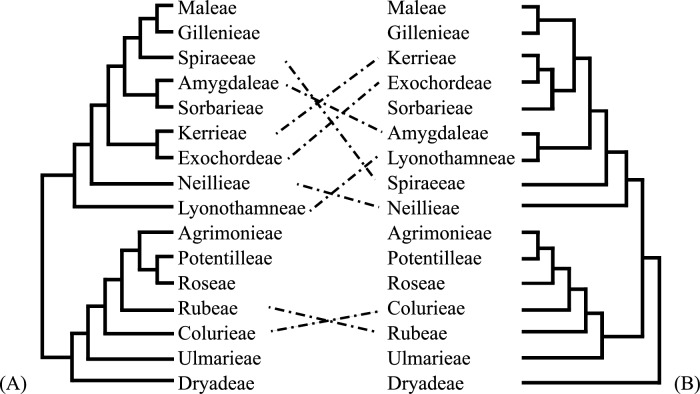
Summary of (**A**) plastome and (**B**) nuclear phylogenies in Rosaceae highlighting incongruencies in tribal relationships. The nuclear phylogeny is a summary tree derived from Xiang et al.^31^.

The phylogenetic analysis places the two *Polylepis* plastomes in a strongly supported clade with the rest of the *Polylepis* accessions, consistent with the clear morphological delimitation of the genus. The most striking morphologically feature of *Polylepis* is its woody habit and distinctive shredding, multilayered bark^8,9^. Other features typical of the genus include the presence of stipular sheaths, imparipinnate leaves, apetalous hermaphroditic flowers, exerted stamens, multilobed styles, and indehiscent achenes enclosing a single carpel with one ovule^8,9^. *Polylepis* is nested in the tribe Agrimonieae, characterized by having 1–5 pistils and achene fruits^32^. Phylogenies derived from plastome and nuclear data show incongruencies in tribal relationships (see Fig. 5). Such discordance has been well-documented and attributed to the nuclear genome’s biparental inheritance patterns reflecting effects of hybridization, introgression, incomplete lineage sorting, duplication, and polyploidy^33–35^, whereas plastome data in Rosaceae represent only the phylogenetic relationships of the maternal lineage.

The complete plastomes for *Polylepis* provided here lay the groundwork for broader phylogenomic studies across the genus, particularly in comparative genomics to test the species boundaries proposed by Espinoza & Kessler^8^. Additionally, a backbone phylogeny generated from plastome data in comparison with nuclear data will allow evaluation of hybridization events and evolutionary trajectories in *Polylepis*. This information will provide the scientific basis for understanding how the genus has evolved physiological adaptations to thrive in some of the most challenging environments inhabited by trees, characterized by sub-zero nocturnal temperatures, extreme diurnal temperature variations of up to 50 °C, high UV radiation, and strong drought^36–39^. For instance, it has been hypothesized that these evolutionary adaptations are promoted by gene exchange via hybridization as well as by polyploidization^20,40,41^. Understanding these evolutionary patterns will allow informed conservation strategies to protect the genetic diversity and adaptive potential of *Polylepis* species in the Andes by guiding restoration efforts and designation of priority areas for conservation, ensuring the long-term survival of these ecologically and culturally unique trees^17,42,43^.

## Methods

### Study species

*Polylepis australis* and *P. microphylla* were sampled for this study. *Polylepis australis* (sect. *Australes sensu* Boza Espinoza & Kessler^8^) is endemic to Argentina, distributed from Jujuy Province to Córdoba Province^8^. It grows on humid subtropical mountains as well as in dry forests, at elevations ranging from 1230 m to 3800 m. This species is distinguished by its glabrous leaflets (sometimes the abaxial surfaces are sparsely hispid) and its winged fruits^8^. *Polylepis microphylla* (sect. *Reticulatae sensu* Boza Espinoza & Kessler^8^) occurs in small, isolated populations in the central Ecuadorian Andes on the slopes of Volcán Chimborazo, in north-western Peru in the high Andes of Cajamarca, the Cordillera Blanca and the adjacent Cordillera Huayhuash at the boundaries of Ancash and Lima and in Arequipa and Cusco. It mainly grows in arid zones at 3150–4550 m elevation^8^. Morphologically it is similar to *P. simpsoniae* T.Boza & M.Kessler but differs in its smaller leaflet size (0.3–0.7 × 0.2–0.5 cm versus 0.9–1.6 × 0.4–1.1 cm), longer leaflet hairs (0.8–1.0 mm versus 0.5–0.7 mm) and inflorescences with fewer flowers (1–3 versus 3–5). Kessler et al.^44^ reported multiple ploidy levels in *P. australis* based on flow cytometry and Boza Espinoza et al.^45^ reported multiple ploidy levels in *P. microphylla* based on guard cell size measurements. *Polylepis australis* was collected from Sierras Grandes de Córdoba, Los Condoritos, Argentina (31° 28′ 59″ S, 64° 49′ 59″ W) and *P. microphylla* was collected from Chacan, Peru (13° 29’ 04" S, 71° 59’ 26" W). Collection and DNA extraction permits were granted through permit numbers: R.D.G. N° 233-2015, N° 237-2015-SERFOR/

DGGSPFFS, MAE-DNB-CM-2018-0082, and MAAE-DBI-CM–2021–0171. Formal identification of the samples was done by M. Kessler who published the most recent taxonomic monograph of *Polylepis*^8^.

### Sample collection, DNA Extraction, library preparation and sequencing

Leaf material from *Polylepis australis* (*M. Lehnert 4112,* HAL 00168271) and *P. microphylla* (*M. Lehnert 4113,* HAL 00168272) cultivated at Halle Botanical Garden was sampled and silica-gel dried. Genomic DNA from 20 mg dried material was extracted using Macherey-Nagel NucleoSpin Plant II-Kit following the manufacturer’s protocol with additional sorbitol pre-wash. DNA quality and fragment length were checked on a 1.5% agarose gel and DNA concentration and purity were estimated on a Qubit fluorometer. Extracts were then sent to Macrogen Europe for library preparation and sequencing on a NovaSeqX platform.

### Illumina read processing and annotation

Illumina reads were *de novo*-assembled using the software GetOrganelle vers. 1.7.7.1^46^ with the following parameters: database set to embplant_pt, k-mer size ranged from 21 to 105, maximum number of extension rounds set to 30, and the maximum number of reads set to 7.5e7. The resulting plastome was annotated using the online platform GeSeq^47^ (available at https://chlorobox.mpimp-golm.mpg.de/geseq.html) setting *Sanguisorba officinalis* (NCBI RefSeq NC_044694.1) as the reference. BLAT^48^ searches were done by setting the protein search identity to 80% and the rRNA, tRNA, and DNA search identity to 85%. Chloroplast CDS and rRNAs annotations were done using the MPIMP land plant references and tRNA annotation was done through a HMMER profile search on ARAGORN vers. 1.2.38^49^. Annotations were checked manually on Geneious v.8.1.9^50^. The final annotated plastomes were converted into graphical maps using OGDRAW^51^ (available at https://chlorobox.mpimp-golm.mpg.de/OGDraw.html) using default settings. Robustness of the assembled plastomes was assessed using PACVr (Plastome Assembly Coverage Visualization in R)^27^.

### Hypervariable site identification

The two assembled plastomes were aligned using MAFFT^52^. Nucleotide diversity (π) was assessed to identify hypervariable regions in the assembled plastomes of *Polylepis* using a sliding window analysis in DnaSP v6^28^. Window length was set to 600 bp and step size set to 200 bp comparable to other Rosaceae plastome studies^29,30^.

### Phylogenetic analysis

The correct phylogenetic position of the two assembled plastomes in the family Rosaceae was evaluated using Maximum Likelihood (ML) analysis. Accessions of plastome sequences utilized in the analysis are found on the complete ML tree in Supplementary Figure 3. Sequences were aligned using MAFFT^52^. The final alignment consisted of 242,422 base pairs. ML analysis was performed on IQ-TREE multicore version 2.4.0^53^ with ultrafast bootstrap (UFBoot)^54^ of 1000 replicates. ModelFinder^55^ was used to identify the best-fitting model of sequence evolution based on the Bayesian Information Criterion (BIC), which selected TVM+F+I+R9.

## Supplementary Information

Supplementary Information.

## Data Availability

Raw sequence reads and annotated plastomes are available on European Nucleotide Archive (ENA) under project accession number PRJEB89853. Voucher specimens are deposited at Martin-Luther-Universität Halle-Wittenberg Herbarium (HAL) with accession numbers HAL 00168271 (*P. australis*) and HAL 00168272 (*P. microphylla*).
