# Supplementary material for: First complete plastomes of the emblematic Andean tree genus Polylepis Ruiz & Pav. (Rosaceae)
Source: Sci Rep. 2025 Sep 23;15:32681. doi: 10.1038/s41598-025-20603-8 (PMC12457594; doi:10.1038/s41598-025-20603-8)

Supplementary Table 1. Gene features of the complete *P. australis* and *P. microphylla* plastomes.

**Gene Category**	***P. australis***	***P. microphylla***
**Protein-coding genes**	81	81
**tRNA genes**	32	32
**rRNA genes**	4	4
**Duplicated in IR**	17	17
**Intron-containing**	18	18
**Putative pseudogenes**	1	1

Supplementary Table 2. Percent similarity of *Polylepis* plastomes.

	**Pmicro**	**Paustra**	**P_australis KY419989.1**	**Polylepis_sp KY419992.1**	**Polylepis_reticulata KY419921.1**	**Polylepis_rugulosa OQ834952.1**
**Pmicro**		99.9	99.2	99.2	99.2	98.9
**Paustra**	99.9		99.2	99.3	99.2	98.9
**P_australis_KY419989.1**	99.2	99.2		99.9	99.3	99.1
**Polylepis_sp_KY419992.1**	99.2	99.3	99.9		99.4	99.2
**Polylepis_reticulata_KY419921.1**	99.2	99.2	99.3	99.4		98.8
**Polylepis_rugulosa_OQ834952.1**	98.9	98.9	99.1	99.2	98.8	

Supplementary Figure 1. Results of the MAUVE alignment showing conserved platome structure in the *Polylepis* accessions.

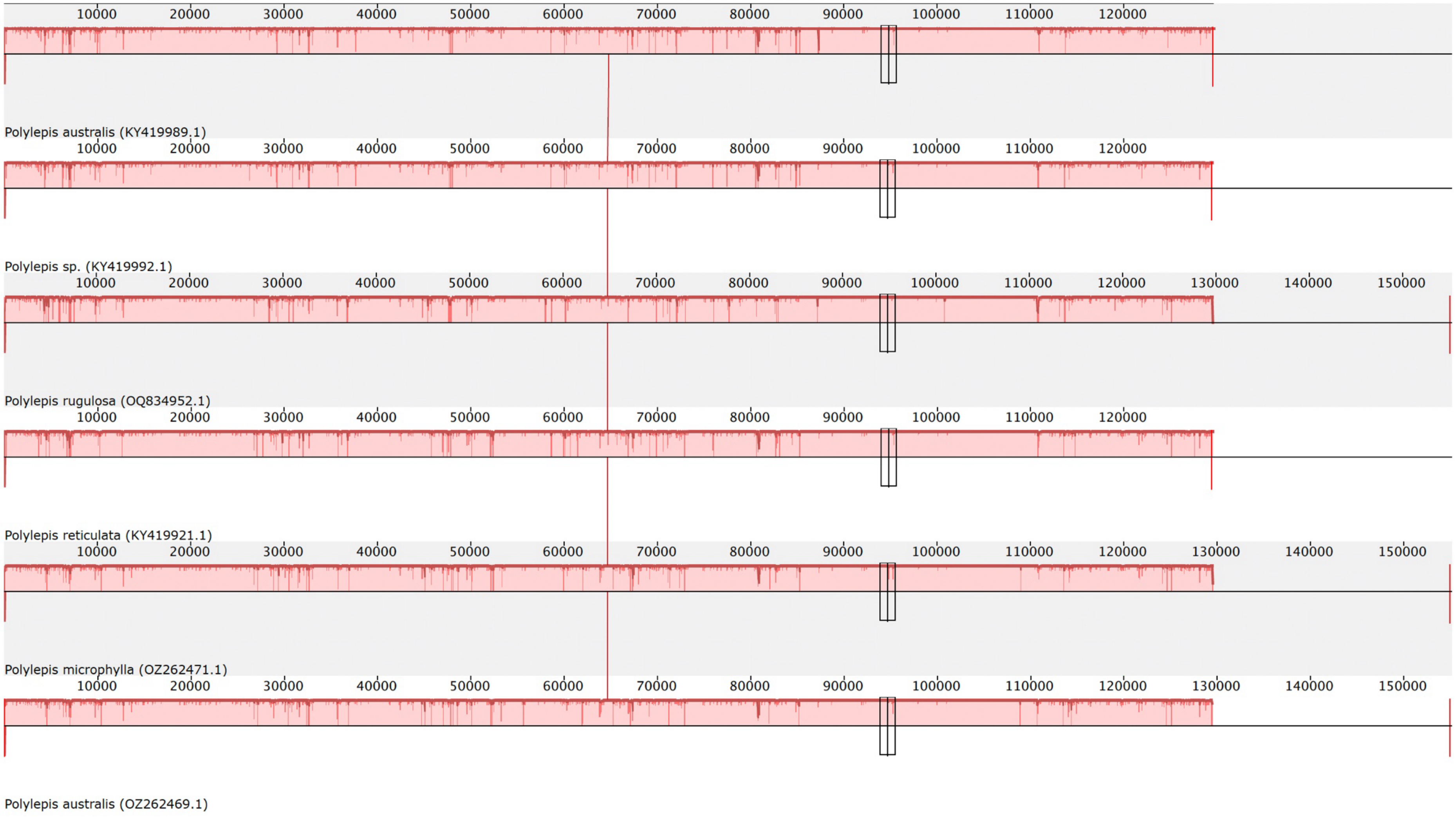

Supplementary Figure 2. Comparison of the *P. australis* partial plastome (top) and complete plastome (bottom). Black bars indicate repeat insertions, red bars mark SNPs, and the blue bar denotes the missing IR region in the partial plastome.

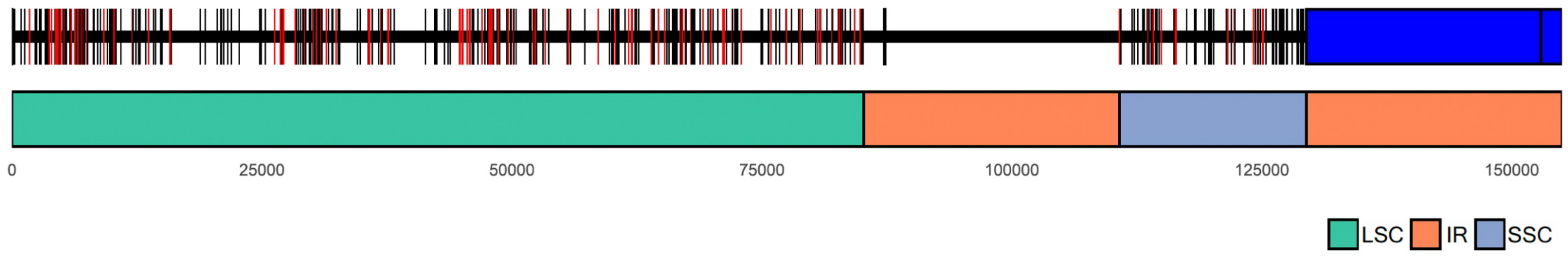

Supplementary Figure 3. Maximum Likelihood (ML) tree of Rosaceae plastomes.

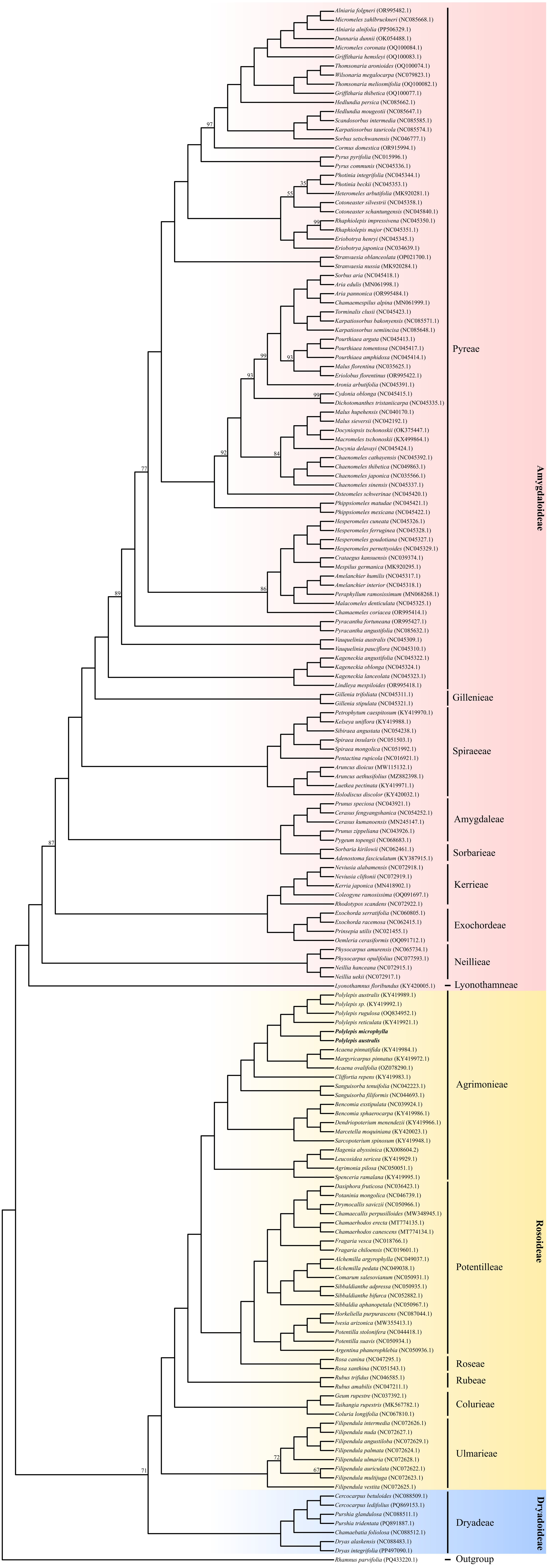

Supplementary Figure 4. Maximum Likelihood (ML) tree of Polylepis accessions.

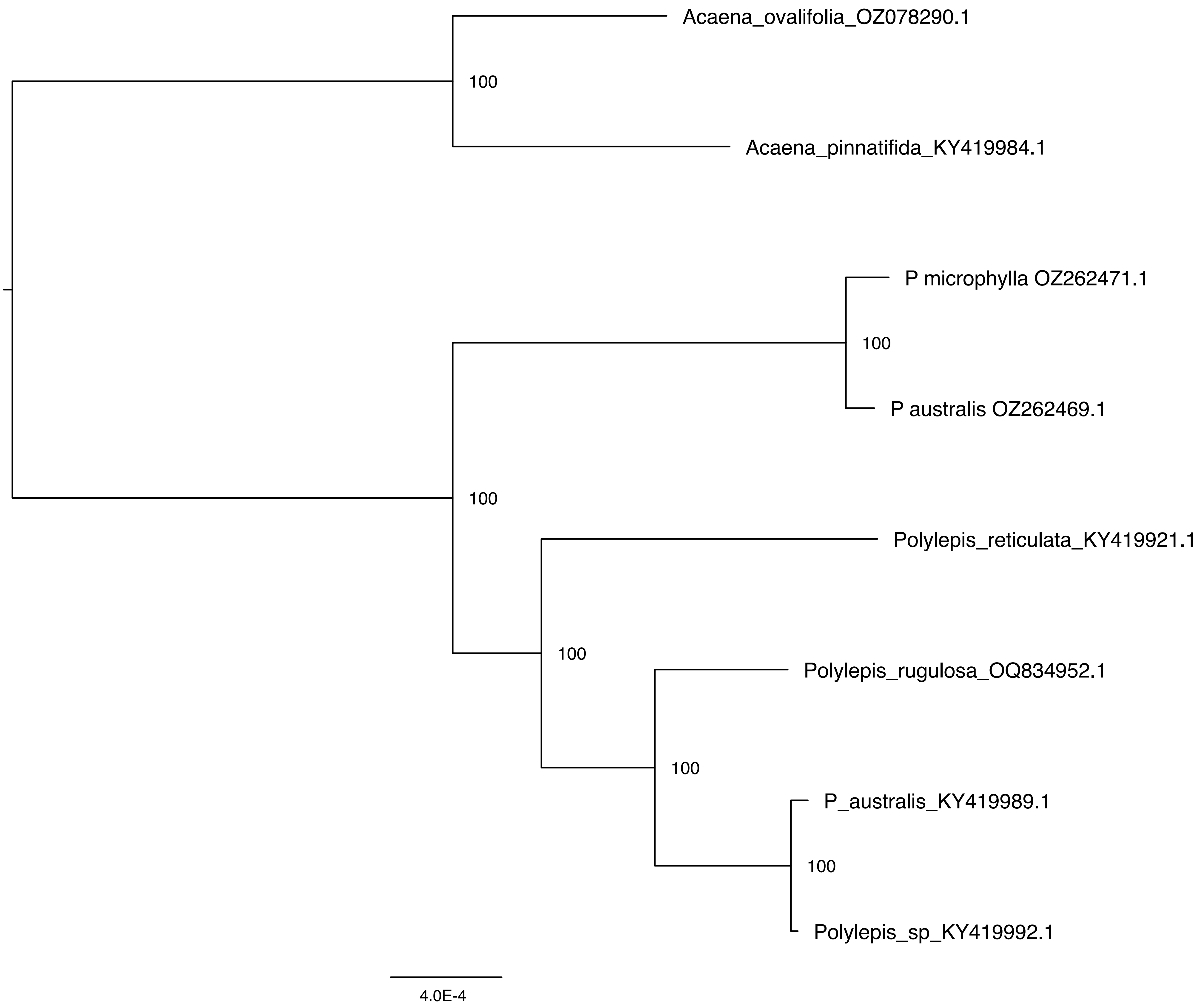

Supplement: Supplementary file 1 — Supplementary Information. [file 41598_2025_20603_MOESM1_ESM.pdf]
